# P-2079. A Retrospective Analysis of the Reproducibility of Discordant Fourth-Generation Quantiferon Tests

**DOI:** 10.1093/ofid/ofae631.2235

**Published:** 2025-01-29

**Authors:** Arun Burra, Christopher J Graber, Matthew B Goetz, Kevin Ikuta

**Affiliations:** David Geffen School of Medicine at UCLA, Santa Monica, California; VA Greater Los Angeles Healthcare System/UCLA, Los Angeles, California; VA Greater Los Angeles Healthcare System, Los Angeles, California; West Los Angeles VA, Los Angeles, California

## Abstract

**Background:**

Interferon-gamma release assays (IGRAs) are used for tuberculosis (TB) screening. The Quantiferon-TB Gold Plus (QFT-Plus) assesses T-cell reactivity to two distinct sets of TB antigens; the test is considered positive if either assay returns positive. Many studies suggest retesting patients with low level positivity prior to proceeding with TB treatment given high rates of reversion in this group. The significance and reproducibility of when TB antigen minus nil (TB Ag-Nil) in one assay is positive and the other is negative (i.e., a discordant result) is unknown.

**Figure:**
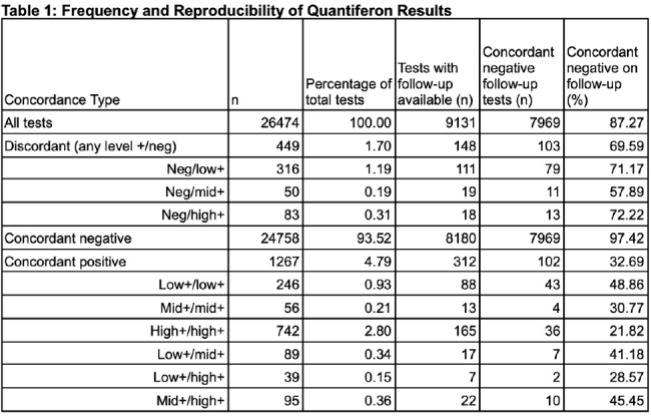

**Methods:**

We reviewed 26,474 QFT-Plus tests completed for any purpose between 2018 and 2024 at an academic VA medical center. Excluding indeterminate tests, we noted the frequency and (where follow-up tests were available) reproducibility of discordant results. Based on a literature review on the reproducibility of prior generation tests, we defined levels of positivity: “low+” TB Ag-Nil as 0.35-0.69 IU/mL, “mid+” 0.7-0.99 IU/mL, and ”high+” ≥1 IU/mL. For statistical analysis, we performed univariate logistic regression assessing the probability of follow up QFT testing returning negative (reversion) in a discordant result compared to a concordant low+ result.

**Figure:**
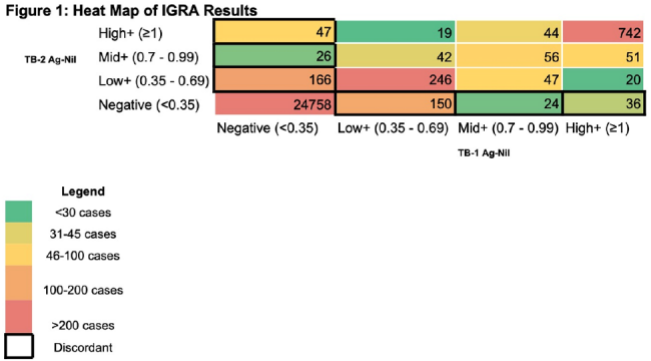

**Results:**

During the study period, 449 QFT-Plus test results were discordant, representing 26% of all positive QFT-Plus tests (Table 1). Among all discordant tests with follow-up available, 70% (103/148) reverted to negative while 49% (43/88) of repeated concordant low+ tests reverted. Discordant tests were more likely to revert than were concordant low+ tests (OR 2.40, 95% CI 1.39-4.15, p=0.002). 71% (79/111), 58% (11/19), and 72% (13/18) of repeat tests were negative with an initial discordant test that was low+, mid+, or high+, respectively (Table 1). Most discordant results were low+ (Figure 1).

**Conclusion:**

Upon retesting, discordant QFT-Plus tests most often revert. The level of positivity in discordant tests does not appear to correspond with the likelihood of reversion. We found that the rates of reversion for discordant tests were significantly higher than for concordant lowly positive tests, suggesting a role for retesting patients with discordant results — regardless of the actual test values — prior to decision making around TB treatment in some settings.

**Disclosures:**

All Authors: No reported disclosures

